# More intensive hepatitis C virus care models promote adherence among people who inject drugs with active drug use: The PREVAIL study

**DOI:** 10.1111/jvh.13756

**Published:** 2022-10-12

**Authors:** Moonseong Heo, Irene Pericot‐Valverde, Jiajing Niu, Brianna L. Norton, Matthew J. Akiyama, Shadi Nahvi, Julia H. Arnsten, Alain H. Litwin

**Affiliations:** ^1^ Department of Public Health Sciences Clemson University Clemson South Carolina USA; ^2^ Department of Psychology Clemson University Clemson South Carolina USA; ^3^ School of Mathematical and Statistical Sciences Clemson University Clemson South Carolina USA; ^4^ Division of General Internal Medicine, Department of Medicine Albert Einstein College of Medicine Bronx New York USA; ^5^ Department of Medicine University of South Carolina School of Medicine Greenville South Carolina USA; ^6^ Department of Internal Medicine Prisma Health Greenville South Carolina USA; ^7^ Clemson University School of Health Research Clemson University Clemson South Carolina USA

**Keywords:** adherence, DAA, drug use, HCV, PWID

**Abbreviations**
DAA: direct‐acting antiviral
DTF: daily time frame
GT: groups therapy
HCV: hepatitis C infection
mDOT: modified directly observed therapy
OTP: opioid treatment program
PWID: people who inject drugs
SIT: standard individual therapy
SVR: sustained virologic response

## 1 INTRODUCTION

Direct‐acting antiviral (DAA) therapies are more effective for treating hepatitis C virus (HCV) with higher rates of sustained virologic response (SVR) and fewer side effects than older interferon‐based treatments.^1^ DAAs are effective even among people who inject drugs (PWID),^2^ a population disproportionately infected by HCV.^3^ Nonetheless, adequate adherence is crucial for PWID living with HCV to achieve SVR and reduce transmission, a public health goal which has been prioritized for global efforts to eliminate HCV by 2030.^4^ How ongoing drug use interferes with HCV treatment adherence support remains unknown.^5^, ^6^

The PREVAIL study^2^ was conducted in opioid treatment program (OTP) settings to test the effectiveness of three models of HCV care for PWID. The trial showed that higher adherence was associated with successful SVR and the adherence level was highest among participants treated with modified directly observed therapy (mDOT) compared to those treated with groups therapy (GT) or standard individual therapy (SIT).^7^ We examined whether more intensive care models such as mDOT or GT would also increase adherence even among participants with active drug use compared to those without at baseline and during treatment.

## 2 METHODS

### 2.1 Setting and design

We performed a secondary analysis of data from the PREVAIL study that randomized three HCV care models—SIT, GT and mDOT—with a 1:1:1 randomized allocation ratio. The study was conducted at three OTP clinics that offer on‐site medical care in the Bronx, NY. Electronic blister packs were used to record the time and date of each opening for a medication retrieval.^8^

### 2.2 HCV treatments

Treatment regimens included *combination DAA* treatments of sofosbuvir/ledipasvir or sofosbuvir/simeprevir, and *interferon‐containing* treatments of telaprevir/pegylated interferon/ribavirin, sofosbuvir/pegylated interferon/ribavirin or sofosbuvir/ribavirin.

### 2.3 Participants

A total of *N* = 150 PWID were enrolled. We analysed data from *N* = 147 participants who returned at least one blister pack. Baseline characteristics stratified by treatment regimens are provided in Table S1.

### 2.4 Drug use

Urine toxicology tests were conducted at baseline and at Weeks 4, 8 and 12 research visits during the treatment period. Active drug use was defined by a urine toxicology being positive for amphetamine, benzodiazepine, cocaine, opiate or oxycodone. The distribution of use of each drug during the research visits is provided in Table S2. We considered four binary (yes vs. no) drug use indicator measures with respect to whether or not participants: (1) used any drug at baseline; (2) ever used any drug during treatment with one or more visits with a positive result; (3) frequently used any drug during treatment with two or more visits with a positive result; and (4) concurrently used any drug in a time‐by‐time fashion over the three visits during treatment. The first three measures are cross‐sectional whereas the last is longitudinal.

### 2.5 Adherence

Adherence for every treatment day was determined based on the blister pack opening date, and daily time frame (DTF) window was set between 12:00 am to 11:59 pm. DTF adherence was defined as DTF = 0 for no opening, and DTF = 1 for one or more openings on a given treatment day. Undetermined DTF adherence on missing dates due to lost or unreturned blister pack (<4%) were treated as missing. DTF was converted to three monthly percentages of adherence to align with the urine toxicology test time frame. Although the prescribed treatment weeks varied (8, 12 or 24 weeks), the medications were dispensed in the electronic blister packs only up to 12 weeks.

### 2.6 Covariates

Baseline potential confounding clinical factors included cirrhosis status, IL28B genotypes, HIV co‐infection, psychiatric illness (any major depressive episode, psychotic disorder, generalized anxiety disorder or current manic episode by medical chart review) and alcohol intoxication for one or more days within 30 days prior to baseline.

### 2.7 Statistical analysis

Mixed‐effects linear models were applied to compare *adjusted* adherence levels between participants with and without drug use by treatment arm in the form of Monthly Adherence = Arm + DrugUse + Time + Arm*DrugUse + Covariates, where the variable DrugUse was a binary drug use measure. Unadjusted *crude* adherence levels were compared using the mixed‐effect models without covariates. These modelling frameworks were used with each of the four drug use definitions. The arm‐by‐drug use interaction term, Arm*DrugUse, was the primary parameter of interest. What followed was post hoc testing of significance of difference in adherence, estimated based on the model, between participants with and without drug use for each treatment arm. As sensitivity analysis, all analyses were stratified by the two types of medication regimens, combination DAA (*N* = 115) and interferon‐containing treatments (*N* = 32). Adjusted estimates are first reported, followed by collective results of crude estimates and stratified analysis. All statistical analyses were performed using SAS v9.4 (SAS Inc.), and statistical significance was declared if a two‐sided *p*‐value < .05.

## 3 RESULTS

### 3.1 Impact of baseline drug use on adherence

**FIGURE 1:**
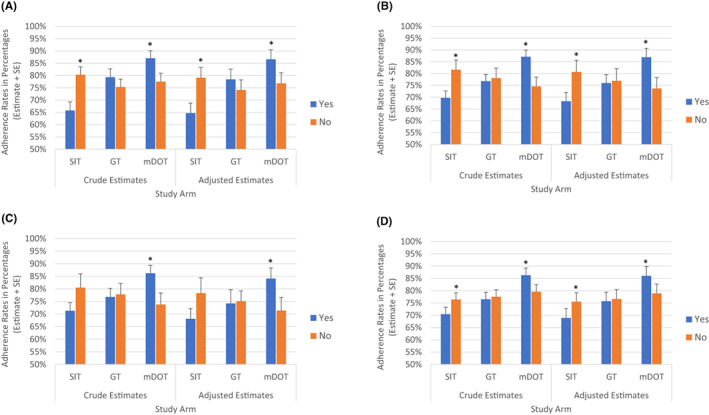
Adherence level between drug use adherence by study arms (*N* = 147; *n*(SIT) = 48, *n*(GT) = 48, *n*(mDOT) = 51). The *adjusted* adherence level and its standard error (SE) were estimated from mixed‐effects linear after adjusting for IL28b, HIV status, Cirrhosis, psychiatric illness and alcohol intoxication whereas *crude* adherence levels were estimated without any covariate adjustments. The binary measures of the active drug use were as follows: (A) drug use at baseline with a positive result (crude interaction *p* = .001, adjusted interaction *p* = .001); (B) ever drug use at any visit during treatment period with one or more visits with a positive result (crude interaction *p* = .003, adjusted interaction *p* = .002); (C) frequent drug use during treatment with two or more visits with a positive result (crude interaction *p* = .037, adjusted interaction *p* = .024); and (D) longitudinal concurrent drug uses over the three visits during treatment period (crude interaction *p* = .016, adjusted interaction *p* = .009). Significant differences (*p* < .05) were denoted by *.

Differences in adherence between participants with and without baseline drug use were significantly different across the three arms (*p* = .001). Specifically, adherence was significantly higher for participants with baseline drug use than those without in mDOT (86.6 ± 3.9 [SE] vs. 76.8 ± 4.3, *p* = .035), but adherence was significantly lower for those with baseline drug use in SIT (64.7 ± 4.1 vs. 79.1 ± 4.2, *p* = .003; Figure 1A).

### 3.2 Impact of ever drug use during treatment

Differences in adherence between participants with and without any time drug use during treatment were significantly different across the three arms (*p* = .002). Adherence was significantly higher for participants with any time active drug use during treatment than those without in mDOT (86.9 ± 3.8 vs. 73.7 ± 4.6, *p* = .007) but it was significantly lower for those with baseline drug use in SIT (68.3 ± 3.8 vs. 80.7 ± 4.9, *p* = .017; Figure 1B).

### 3.3 Impact of frequent drug use during treatment

Differences in adherence between participants with and without time frequent drug use during treatment were significantly different across the three arms (*p* = .024). Adherence was significantly higher for participants with frequent active drug use during treatment than those without in mDOT (84.1 ± 4.3 vs. 71.4 ± 5.2, *p* = .024; Figure 1C).

### 3.4 Impact of concurrent drug use during treatment

Differences in adherence between participants with and with contemporaneous drug use time during treatment were significantly different across the three arms (*p* = .009). Adherence was significantly higher for participants with concurrent drug use during treatment than those without in mDOT (86.0 ± 3.9 vs. 78.9 ± 3.8, *p* = .029) whereas it was significantly lower for those with concurrent drug use in SIT (68.9 ± 3.8 vs. 75.5 ± 3.7, *p* = .030; Figure 1D).

### 3.5 Crude estimates and stratified analysis results

There were little differences between the crude and the adjusted estimates across entire analyses (Figures 1, S1 and S2). The overall findings presented in Figure 1 are fairly consistent regardless of the types of treatment regimens between combination DAA and interferon‐containing treatments (Figures S1 and S2, respectively); however, drug users in GT group treated with interferon‐containing medications had higher adherence compared to their GT counterparts for baseline drug users, and ever and frequent users during treatment (Figure S2).

## 4 DISCUSSION

We found that the impact of active drug use, at baseline or during treatment, on DAA adherence was significantly different across three HCV care models with varying levels of intensity of care. Regardless of how active drug use was defined, we found that the HCV care model was significantly associated with differences in adherence between participants with and without active drug use. Specifically, GT eliminated the reduction in adherence among those with drug use compared to those without, and mDOT was associated with higher adherence for participants with drug use.

We first observed the following dose–response relationships between adherence and intensity levels of care models. Active drug users had (1) the highest adherence level in the mDOT arm, the most intensive care model; (2) the lowest adherence level the SIT arm, the least intensive care model; and (3) middle adherence in the GT arm, the middle level of intensity of care. Second, we found that, compared to those without drug use, participants with drug use had higher adherence in the mDOT arm whereas those in the SIT arm had lower adherence. Lastly, there was no apparent dose–response relationship between adherence and intensity of care among participants without drug use. Somewhat different observations among participants treated with interferon‐containing medications may be due to small sample sizes, but those medications are no longer frequently prescribed in present practices.

The findings imply that more intensive care models may not be necessary for PWID living with HCV without drug use to promote DAA adherence during treatment. However, more intensive care models such as DOT or its modified variants should better be implemented to promote adherence among PWID living with HCV with drug use. Drug screening results at the initiation of medications could serve as a guidance for selecting HCV care modality. Furthermore, regular monitoring of drug use during treatment through self‐report or urine test would also be informative as to whether care modality or intensity level should be changed or adjusted to better support medication adherence.

In sum, more intensive models of care should be implemented to promote adherence and treat HCV among PWID especially with drug use as such models of care might eliminate the negative impact of drug use during treatment on adherence.

## FUNDING INFORMATION

The work was supported in part by grants from the National Institutes of Health (R01DA034086 and K99/R00DA043011) and from Gilead Sciences (IN‐337‐1779).

## CONFLICT OF INTEREST

Dr. Litwin reports grants from Gilead Sciences during the conduct of the study, and grants and personal fees from Gilead Sciences and Merck Pharmaceuticals outside the submitted work. Authors not named here have disclosed no conflicts of interest.

## Supporting information


Appendix S1

## Data Availability

Data sharing requests will be reviewed by the PIs to ensure that the proposed work is both possible and credible, and it meets reasonable demands of scientific integrity. De‐identified data will be shared only with qualified investigators whose research protocols have been approved by their IRB.
