# Supplementary material for: More intensive hepatitis C virus care models promote adherence among people who inject drugs with active drug use: The PREVAIL study
Source: J Viral Hepat. 2022 Oct 12;30(2):172–5. doi: 10.1111/jvh.13756 (PMC9851956; doi:10.1111/jvh.13756)
Supplement: Supplementary file 1 — Appendix S1 [file JVH-30-172-s001.docx]

**Supplementary Table 1**: Baseline Characteristics of the participants

|  | **Mean (SD), n (%)** | | |
| --- | --- | --- | --- |
|  |  | **Treatment Regimen** | |
| **Characteristics** | **All (N=147)** | **DAA combination (N=115)** | **Interferon-Containing (N=32)** |
| Age in years | 51.4(10.7) | 51.4 (10.7) | 51.4 (10.7) |
| Ethnicity |  |  |  |
| Non-Hispanic | 55 (37.4%) | 47 (40.9%) | 8 (25%) |
| Hispanic | 92 (62.6%) | 68 (59.1%) | 24 (75%) |
| Race |  |  |  |
| African American | 40 (27.2%) | 33 (28.7%) | 7 (21.9%) |
| Latino/a | 82 (55.8%) | 62 (53.9%) | 20 (62.5%) |
| White | 12 (8.2%) | 11 (9.6%) | 1 (3.1%) |
| Other | 12 (8.8%) | 9 (7.8%) | 4 (12.5%) |
| Sex |  |  |  |
| Male | 95 (64.6%) | 69 (60.0%) | 26 (81.2%) |
| Female | 52 (35.4%) | 46 (40%) | 6 (18.8%) |
| Cirrhosis |  |  |  |
| Yes | 41 (27.9%) | 32 (27.8%) | 9 (28.1%) |
| No | 106 (72.1%) | 83 (72.2%) | 23 (71.9%) |
| IL28B |  |  |  |
| TC/TT | 115 (78.2%) | 91 (79.1%) | 24 (71.9%) |
| CC | 32 (21.8%) | 24 (20.9%) | 9 (28.1%) |
| Psychiatric Illness |  |  |  |
| Yes | 40 (27.2%) | 35 (30.4%) | 5 (15.6%) |
| No | 107 (72.8%) | 80 (69.6%) | 27 (84.4%) |
| Alcohol Intoxication |  |  |  |
| Yes | 35 (23.8%) | 25 (21.7%) | 10 (31.3%) |
| No | 112 (76.2%) | 90 (78.3%) | 22 (68.7%) |
| HIV |  |  |  |
| Yes | 21 (14.3%) | 14 (12.2%) | 7 (21.9%) |
| No | 126 (85.7%) | 101 (87.8%) | 25 (78.1%) |
| Study Arm |  |  |  |
| SIT | 48 (32.7%) | 39 (33.9%) | 9 (28.1%) |
| GT | 48 (32.7%) | 40 (34.8%) | 8 (25.0%) |
| mDOT | 51 (34.7%) | 36 (31.3%) | 15 (46.9%) |
| Medication Regimens |  |  |  |
| SOF/LDV | 104 (70.8%) | 104 (90.4%) | - |
| SOF/SMV | 11 (7.5%) | 11 (9.6%) | - |
| SOF/RBV | 16 (10.9%) | - | 16 (50.0%) |
| SOF/RBV/PEG | 13 (8.8%) | - | 13 (40.6%) |
| TVR/RBV/PEG | 3 (2.0%) | - | 3 (9.4%) |

Notes: SIT= standard individual therapy; GT= group therapy; mDOT =modified directly observed therapy; SOF/LDV = sofosbuvir/ledipasvir; SOF/SMV = sofosbuvir/simeprevir; SOF/RBV= sofosbuvir/ribavirin; SOF/RBV/PEG= sofosbuvir/pegylated interferon/ribavirin; TVR/RBV/PEG = telaprevir/pegylated interferon/ribavirin;

**Supplementary Table 2**: Drug use ascertained by urine toxicology tests at baseline and during treatment

| **Drug** | **Baseline**  **(N=147)** | **Week 4**  **(N=146)** | **Week 8**  **(N=143)** | **Week 12**  **(N=106)** |
| --- | --- | --- | --- | --- |
| Amphetamine | 0% | 0% | 0% | 0% |
| Benzodiazepine | 15.0% | 16.7% | 14.5% | 15.1% |
| Cocaine | 29.3% | 24.0% | 28.0% | 27.4% |
| Opiate | 23.1% | 26.0% | 29.4% | 28.3% |
| Oxycodone | 2.9% | 5.0% | 7.8% | 3.3% |
| Any | 49.7% | 45.9% | 50.3% | 50.0% |

**Supplementary Figure 1**: The adherence level between drug use adherence by study arms among participants treated with interferon-free DAA combination regimens of SOF/LDV or SOF/SMV (N=115; N(SIT)=39, N(GT)=40, N(DOT)=36).


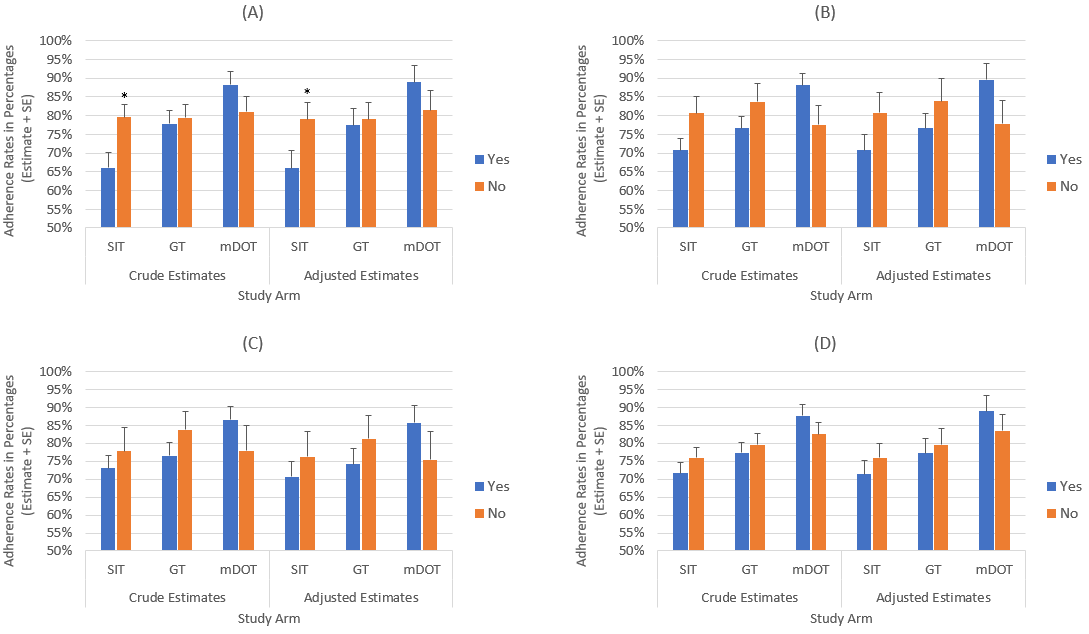


Note: The *adjusted* adherence level and its standard error (se) was estimated from mixed-effects linear after adjusting for IL28b, HIV status, Cirrhosis, psychiatric illness, and alcohol intoxication whereas *crude* adherence levels were estimated without any covariate adjustments. The binary measures of the active drug use were: (A) drug use at baseline with a positive result (crude interaction p=.025, adjusted interaction p=.027); (B) ever drug use at any visit during treatment period with one or more visits with a positive result (crude interaction p=.032, adjusted interaction p=.020); (C) frequent drug use during treatment with two or more visits with a positive result (crude interaction p=.268, adjusted interaction p=.199); and (D) longitudinal concurrent drug uses over the three visits during treatment period (crude interaction p=0.113, adjusted interaction p=.089). Significant differences (p<0.05) were denoted by *.

**Supplementary Figure 2**: The adherence level between drug use adherence by study arms among participants treated with interferon containing DAA regimens of SOF/RBV, SOF/RBV/PEG, or TVR/RBV/PEG (N=32; N(SIT)=9, N(GT)=8, N(DOT)=15).


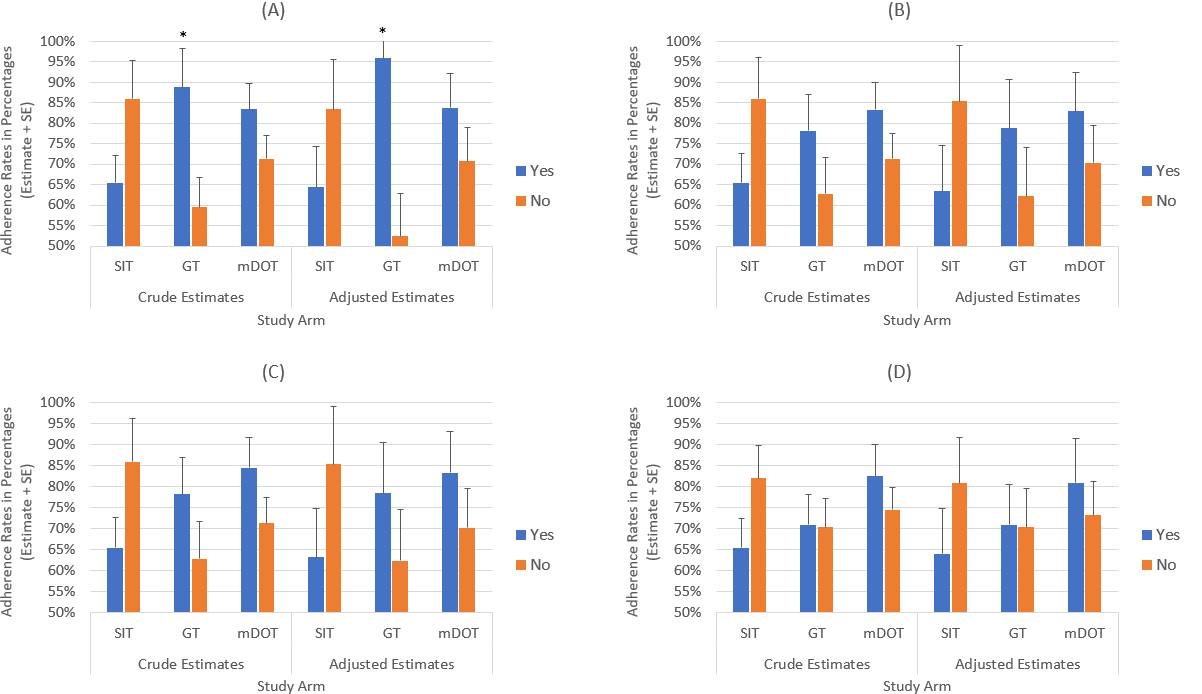


Note: The *adjusted* adherence level and its standard error (se) was estimated from mixed-effects linear after adjusting for IL28b, HIV status, Cirrhosis, psychiatric illness, and alcohol intoxication whereas *crude* adherence levels were estimated without any covariate adjustments. The binary measures of the active drug use were: (A) drug use at baseline with a positive result (crude interaction p=.017, adjusted interaction p=.013); (B) ever drug use at any visit during treatment period with one or more visits with a positive result (crude interaction p=.082, adjusted interaction p=.109); (C) frequent drug use during treatment with two or more visits with a positive result (crude interaction p=.081, adjusted interaction p=.118); and (D) longitudinal concurrent drug uses over the three visits during treatment period (crude interaction p=.136, adjusted interaction p=.178). Significant differences (p<0.05) were denoted by *.
